# Stress-Triggered Long-Distance Communication Leads to Phenotypic Plasticity: The Case of the Early Root Protoxylem Maturation Induced by Leaf Wounding in Arabidopsis

**DOI:** 10.3390/plants7040107

**Published:** 2018-12-04

**Authors:** Ilaria Fraudentali, Renato Alberto Rodrigues-Pousada, Alessandro Volpini, Paraskevi Tavladoraki, Riccardo Angelini, Alessandra Cona

**Affiliations:** 1 Department of Science, University “Roma Tre”, 00146 Rome, Italy; ilaria.fraudentali@uniroma3.it (I.F.); ale.volpini@stud.uniroma3.it (A.V.); paraskevi.tavladoraki@uniroma3.it (P.T.); riccardo.angelini@uniroma3.it (R.A.); 2 Department of Life, Health and Environmental Sciences, University of L’Aquila, 67100 L’Aquila, Italy; pousada@univaq.it

**Keywords:** wounding, root plasticity, hydrogen peroxide, protoxylem

## Abstract

Root architecture and xylem phenotypic plasticity influence crop productivity by affecting water and nutrient uptake, especially under those environmental stress, which limit water supply or imply excessive water losses. Xylem maturation depends on coordinated events of cell wall lignification and developmental programmed cell death (PCD), which could both be triggered by developmental- and/or stress-driven hydrogen peroxide (H_2_O_2_) production. Here, the effect of wounding of the cotyledonary leaf on root protoxylem maturation was explored in *Arabidopsis thaliana* by analysis under Laser Scanning Confocal Microscope (LSCM). Leaf wounding induced early root protoxylem maturation within 3 days from the injury, as after this time protoxylem position was found closer to the tip. The effect of leaf wounding on protoxylem maturation was independent from root growth or meristem size, that did not change after wounding. A strong H_2_O_2_ accumulation was detected in root protoxylem 6 h after leaf wounding. Furthermore, the H_2_O_2_ trap *N*,*N*^1^-dimethylthiourea (DMTU) reversed wound-induced early protoxylem maturation, confirming the need for H_2_O_2_ production in this signaling pathway.

## 1. Introduction

Plant adaptive capacity and acclimatization resources play a pivotal role in increasing plant fitness and survival, especially in fast-changing environmental conditions. Thus, the unravelling of variation in phenotypic plasticity in traits of agronomic interest could provide us with beneficial tools for the development of crops more efficiently adaptable to a changing environment. Phenotypic plasticity integrates genetically determined developmental processes and environmental influences [1], and because of this, identifying phenotypic traits showing favourable adaptive plasticity will provide the basis for further studies focused on assessing the underlying genetic basis.

Root systems play a prominent role in crop health and productivity, especially under resource-limited environmental conditions, and plasticity of root traits, such as root growth and architecture, confers functional adaptivity to soils that are poor in water and nutrients [2]. In this regard, root development and differentiation follow different dynamics and may respond to different signalling pathways under physiological or stress conditions, allowing adaptive plasticity in sub-optimal growth conditions. Under physiological conditions, the boundaries defining the division, elongation and maturation zones of the root are developmentally regulated by the cytokinin/auxin [3,4,5] and/or reactive oxygen species (ROS) pathways [6], and changes in their positions are coordinated with each other [7]. Vascular patterning is finely integrated in the root developmental program by the cytokinin/auxin/thermospermine pathway responsible for the specification of the identity of the protoxylem [8,9], which is going to mature later in the proximal region beyond the zone of maximum elongation growth, where it undertakes the deposition of secondary walls [10].

However, the correlation among root length, meristem size and protoxylem element position may be disrupted under stress or phytotoxic conditions [7], and both ROS [11,12] and stress signalling hormones, such as the wound signal jasmonic acid (JA) [13,14], may assume a role in root length and meristem size specification independently from or interfering with the cytokinin/auxin pathway. In this regard, root xylem phenotypic plasticity has been shown to occur in response to drought stress [15,16], as well as to various stress-simulating conditions [17]. During acclimation to drought, plasticity of root xylem tissues may enhance water absorption from the soil improving plant performance and protecting yield [18]. Moreover, an early xylem differentiation was observed in maize (*Zea mays)*, tobacco (*Nicotiana tabacum*), and Arabidopsis (*Arabidopsis thaliana*) roots under stress-simulated conditions, such as those induced by polyamine (PA)-treatment or amine oxidase (AO)-overexpression [19,20], as well as those signalled by methyl jasmonate (MeJA) treatment [20] or by a compromised status of cell-wall pectin integrity [21]. In these conditions a hydrogen peroxide (H_2_O_2_)-triggered early root xylem maturation, measured as the distance of the first xylem elements with fully developed secondary wall thickenings from the apical meristem, repositions xylem precursors closer to the tip. Furthermore, a higher number of xylem elements in tobacco plants over-expressing a fungal endo-polygalacturonase (PG plants) [21] and in water-stressed soybean has been reported [18]. In the latter system, it has been suggested that this xylem adaptive plasticity enhances water uptake by improving root hydraulic conductivity under drought [18].

Noteworthy, plant dehydration may occur not only under drought, but also as a consequence of those stresses that may lead to excessive water losses, such as leaf mechanical damage caused by herbivore feeding or atmospheric agents. Indeed, it has been reported that several wound-inducible genes were likewise induced by dehydration, implying that water stress is an important component in the plant responses to mechanical wounding [22]. Consistently, other evidence supports the occurrence of cross-tolerance mechanisms between JA-signalled wounding or insect feeding and those stresses that involve perturbation of water potential [23,24]. In this regard, it has been reported that wounding increases salt tolerance in tomato plants [23] and that whitefly infestation promotes drought resistance in maize plants [24], in both cases by a mechanism involving JA biosynthesis [23,24]. In this regard, the phenotypic plasticity of the root xylem system elicited by leaf wounding has never been explored. Here, we provide evidence that wounding of the cotyledonary leaf triggers leaf to root long-distance communication resulting in early root protoxylem differentiation in Arabidopsis. The proposed approach may represent a model for future investigations focused on unravelling the occurrence of phenotypic plasticity induced by long-distance communication triggered by biotic/abiotic stresses imposed at a specific distal site.

## 2. Results

### 2.1. Leaf Wounding Promotes Alteration of Protoxylem Maturation in Root without Affecting Root Length and Meristem Size

**Figure 1:**
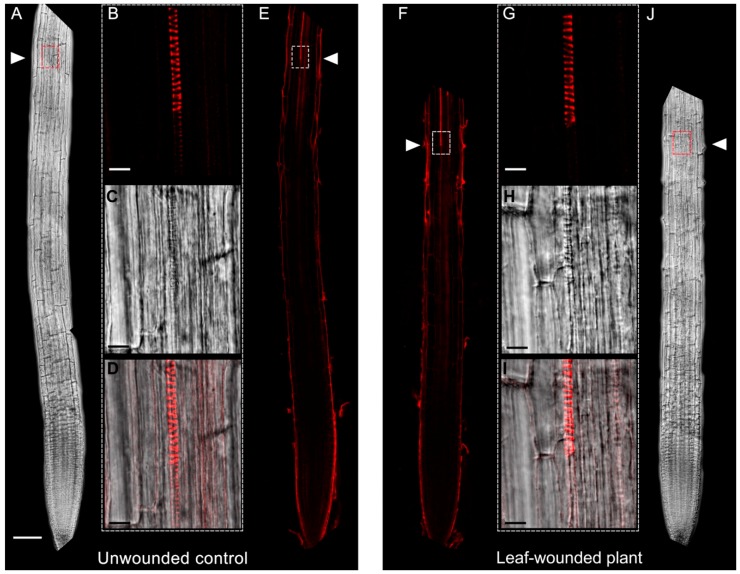
Analysis under LSCM after PI staining of root apexes and respective bright-field images from 10-day-old unwounded control (**A**–**E**) and leaf-wounded (**F**–**J**) seedlings. (**A**–**E**) bright-field of the root from unwounded control seedlings (**A**), PI staining (**B**) bright-field (**C**) and overlay image (**D**) of the magnified zone of the root from unwounded control seedlings, in which protoxylem position (defined by the position of the first protoxylem cell with fully developed secondary cell wall thickenings) is located, PI staining of the root shown in A (**E**); (**F**–**J**) PI staining of the root from leaf-wounded seedling injured at the age of 7 days by cutting the cotyledonary leaf, analysed 3 days after injury (**F**), PI staining (**G**) bright-field (**H**) and overlay image (**I**) of the magnified zone of the root from leaf-wounded seedlings, in which protoxylem position is located, bright-field of the root shown in F (**J**). The images presented are representative of experiments repeated at least five times with ten seedlings analysed each time. Shown images were obtained aligning serial overlapping micrographs of the same root by Photoshop Software (Adobe). Bars: 100 μm (**A**,**E**,**F**,**J**) and 10 μm (**B**–**D**,**G**–**I**).

**Figure 2:**
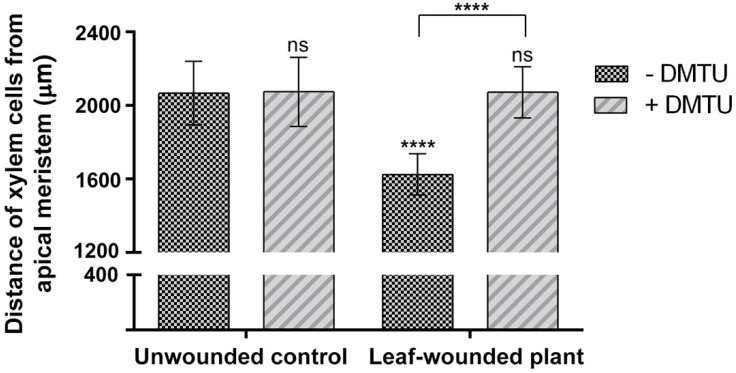
Analysis of differences in protoxylem maturation in leaf-wounded seedlings grown in medium with or without the H_2_O_2_-scavenger DMTU. Distances from the apical meristem of the protoxylem position (defined by the position of the first protoxylem cell with fully developed secondary cell wall thickenings) are reported. These experiments were repeated at least five times with ten seedlings analysed each time (mean values ± SD; *n* = 50). The statistical significance levels between unwounded control DMTU-untreated plants and DMTU-treated and/or wounded plants were evaluated by *p* levels as follows: ****, *p* ≤ 0.0001; ns, not significant. The significance levels between wounded DMTU-untreated and DMTU-treated plants are reported above the horizontal square bracket.

**Figure 3:**
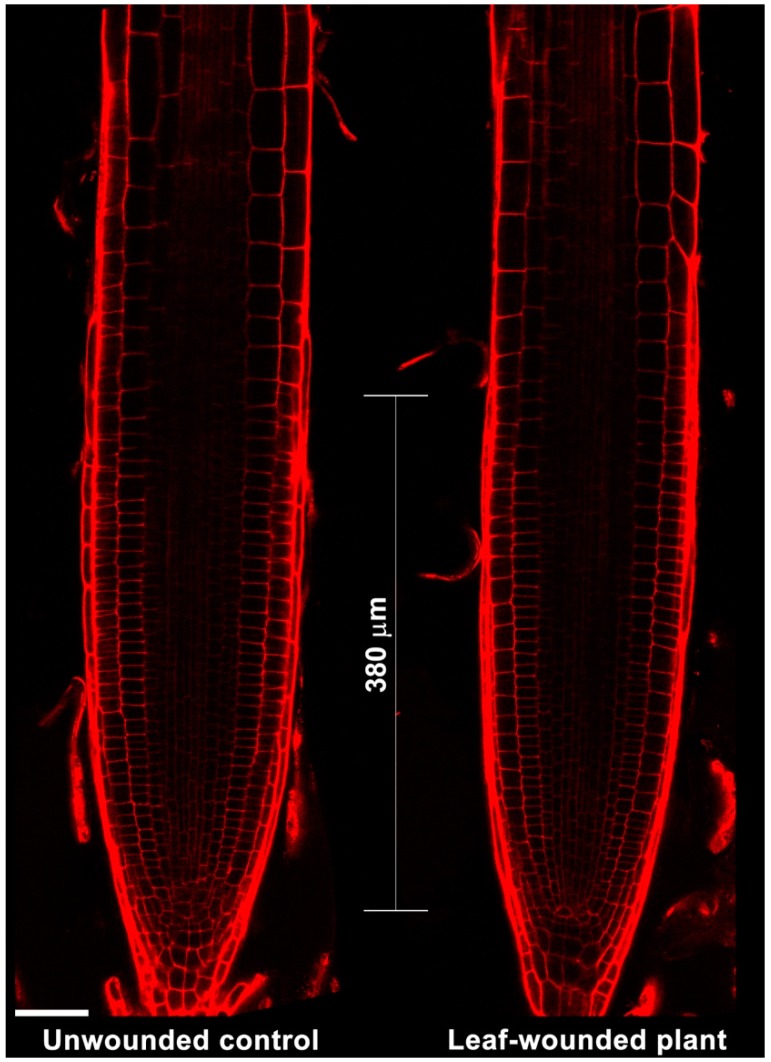
Analysis under LSCM after PI staining of the leaf wounding effect on the length of the meristematic zone, determined by measuring the distance between the quiescent centre and the first elongating cell in the cortex cell file. The images presented show roots from 10-day-old unwounded control and leaf-wounded seedlings, injured at the age of 7 days by cutting the cotyledonary leaf with scissors and analysed 3 days after the injury; roots presented are representative of experiments repeated at least five times with ten seedlings analysed each time. Shown images were obtained aligning serial overlapping micrographs of the same root by Photoshop Software (Adobe). Bar: 50 μm.

To explore the effect of leaf wounding on root xylem phenotypic plasticity, 7-day-old Arabidopsis seedlings were injured by cutting a cotyledonary leaf, and then roots were observed under Laser Scanning Confocal Microscope (LSCM) 3 days after the injury, for the investigation of the distance from the root apical meristem of the first protoxylem cell with fully developed secondary wall thickenings (whose location is here referred as “protoxylem position”) and meristem size. Figure 1 shows images acquired under LSCM after PI staining and relative bright-field images of root apexes from unwounded control and leaf-wounded seedlings. Plantlets in which a cotyledonary leaf was cut present an anticipation of the maturation of protoxylem, as shown by the earlier presence of cells with fully developed secondary wall thickenings that appear closer to the apical meristem as compared to unwounded control plants. Figure 2 demonstrates that these qualitative data were confirmed by statistically significant quantitative analysis. In fact, the mean distance of the first protoxylem cell with fully developed secondary wall thickenings from the root apical meristem was approximately 1620 µm in leaf-wounded plants as compared to unwounded control plants, showing a distance of approximately 2060 µm. The effect of leaf wounding on protoxylem position was specific and not dependent upon variation in root growth or meristem size, which were unchanged in leaf-wounded plants compared to unwounded control plants (Figure 3; Table 1).

### 2.2. Early Xylem Maturation in Arabidopsis Roots upon Leaf Wounding Requires H_2_O_2_

**Figure 4:**
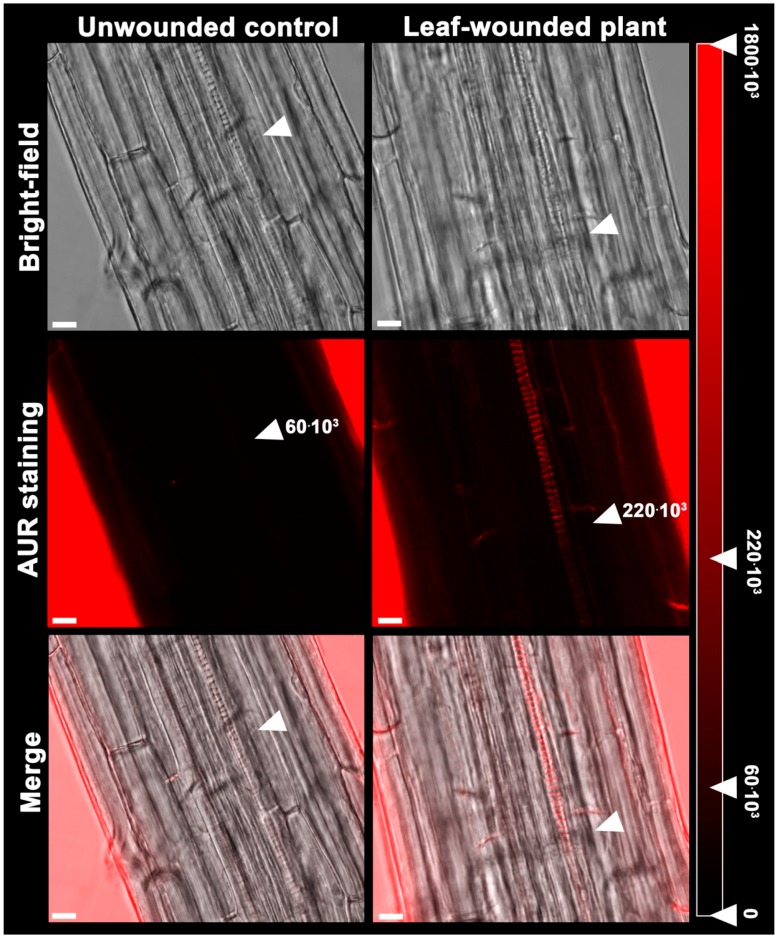
In situ H_2_O_2_ detection by analysis under LSCM after AUR staining of roots from 7-day-old unwounded control and leaf-wounded seedlings 6 h after injury. The corresponding bright-field and overlay images are shown. Micrographs show the root zone corresponding to the site of appearance of the first protoxylem cell with fully developed secondary cell wall thickenings (arrows) and have been taken at the level of the central root section. Images are representative of those obtained from ten seedlings from five independent experiments. In the red degrading scale, the average values of fluorescence intensity, measured as the sum of the pixels of each 65 µm^2^ rectangle, are indicated for unwounded control and leaf-wounded plants, and these were 60 × 10^3^ ± 19 × 10^3^ and 220 × 10^3^ ± 38 × 10^3^, respectively (mean values ± SD; *n* = 25). The maximum pixel sum for a completely saturated square was approximately 1800 × 10^3^. Bar: 10 μm.

Figure 2 also shows that the H_2_O_2_-scavenger *N*,*N*^1^-dimethylthiourea (DMTU), provided at the working concentration of 100 μM, according to a previous report [25], opposes the effect of leaf wounding on early protoxylem maturation consistently with what was previously demonstrated for the MeJA-mediated induction of protoxylem differentiation [20]. To confirm that the effect of the wound-induced signalling on the early maturation of protoxylem cells require H_2_O_2_, this compound was detected in situ in Arabidopsis roots following leaf wounding by exploiting the fluorogenic peroxidase substrate Amplex Ultra Red (AUR). Figure 4 shows that 6 h after leaf wounding, a strong AUR signal was revealed in the root zone where the first protoxylem cell with fully developed secondary cell wall thickenings is found, which was not detectable in unwounded control roots, which is suggestive of a tissue-specific H_2_O_2_ production triggered by a long-distance leaf-to-root communication and leading to early protoxylem differentiation.

## 3. Discussion

Leaf-to-root long-distance communication is crucial in coordinating biochemical and physiological events between aerial and underground organs, especially in response to changes in environmental conditions [26,27,28,29]. Leaf damage is a frequent injury during the plant lifespan, and may be caused by both herbivores, such as chewing insects, and atmospheric conditions. The wound site is an easy passage for both pathogen entry and water loss, and the presence of leaf mechanical damage triggers several local responses devoted to healing the wound [21,30,31,32]. Furthermore, complex signalling networks propagate information from the wound site through the whole plant body, allowing systemic responses [29], among which, xylem root remodelling could represent a strategy for enhancing water uptake and counteracting the excessive water loss caused by the wound.

The analysis of root growth, protoxylem position and meristem size in plants in which the cotyledonary leaf has been cut shows a DMTU-reversible early protoxylem differentiation occurring 3 days after injury (Figure 1 and Figure 2), which is independent from variation in meristem size and root growth, which were unchanged (Table 1; Figure 3). A root protoxylem-specific accumulation of H_2_O_2_ was detectable 6 h after the injury, supporting its involvement in the variation of protoxylem position (Figure 4). This response is consistent with previous data, where roots of MeJA-treated plants showed a H_2_O_2_-dependent remodelling of the protoxylem, which appeared to be closer to the root tip, independent of root growth or meristem size [20]. Based on the effects of MeJA treatment on protoxylem differentiation, it has been hypothesized that under stress conditions, extracellular H_2_O_2_ production may drive early xylem differentiation independently from the auxin/cytokinin/T-Spm loop [17]. In particular, in differentiating protoxylem elements, the H_2_O_2_ production driven by cell wall-localized oxidation of PAs was suggested to be involved in both developmental programmed cell death (PCD) and peroxidase-mediated lignin polymerization [17,19,20], which represent key steps in the terminal phase of the xylem differentiation process. PAs are oxidized to aminoaldehydes by AOs, which include copper-containing amine oxidases (CuAOs) and flavin adenine dinucleotide (FAD)-dependent polyamine oxidases (PAOs), with the production of a corresponding amine moiety and the biologically active compound H_2_O_2_ [33]. Among the cell-wall sources of ROS, it has been known for a long time that AOs are involved in wound-healing responses [30,33] and root xylem differentiation [19,20,21]. Our results suggest the occurrence of a systemic signalling linking an abiotic stress such as leaf wounding with distal root phenotypic plasticity such as variation in protoxylem position, and open the question of unravelling the responsible ROS source.

## 4. Materials and Methods

### 4.1. Plant Materials, Treatments and Root Growth Analysis

Arabidopsis seedlings (Columbia-0 ecotype) were grown in vitro in a growth chamber at 23 °C and 55% relative humidity under a photoperiod of 16 h light and 8 h dark. Sterilization of seeds was carried out according to Valvekens et al. [34]. After cold stratification at 4 °C, seeds were grown in one-half-strength Murashige and Skoog salt mixture added with 0.5% (*w*/*v*) sucrose and 0.8% (*w*/*v*) agar. Plates were kept in vertical position to allow root growth on the solid medium surface. For analysis under LSCM of root protoxylem position and meristem size, 7-day-old seedlings were selected for homogeneity in root length and then transferred onto fresh medium with or without 100 μM DMTU. After the transfer, seedlings were injured by cutting the cotyledonary leaf with scissors, and after 6 h (AUR staining) or 3 days (PI staining), they were collected for analysis under LSCM. The effect of leaf wounding on root growth was evaluated as the difference between the length measured at the onset of the wounding and that measured after 3 days. 

### 4.2. Protoxylem Position and Meristem Size Analysis under LSCM by Cell Wall PI Staining and Bright-Field Examination of Root Tissues

Root apices from 10-day-old unwounded control and leaf-wounded seedlings treated or not with 100 µM DMTU for the last 3 days, were incubated for 5/10 min in PI (10 μg mL^−1^) to highlight cell wall and protoxylem [35] and then observed under LSCM using a 488 nm argon laser, with a 600–680 nm band-pass filter and a 40× oil immersion objective. The PI staining was allowed to proceed until protoxylem was completely highlighted. Roots were concurrently analysed by bright-field microscopy, using the same laser beam as described above. To analyse protoxylem maturation, the distance from the root apical meristem of the first protoxylem cell with fully developed secondary wall thickenings was measured following the method described by Ghuge et al. [20] considering the point where a sharp intensification of protoxylem PI staining was detectable as indicative of fully differentiated secondary cell wall thickenings (this point is referred to here as the protoxylem position). Analysis of protoxylem position was validated by the correspondence between the site where the sharp increase in the PI-induced fluorescence was revealed under LSCM and that of protoxylem appearance under bright-field microscope [20]. The length of the meristematic zone was determined by measuring the distance between the quiescent centre and the first elongating cell in the cortex cell file [13,36,37]. The images shown were obtained by aligning serial overlapping micrographs of the same root using Photoshop Software (Adobe, San Jose, CA, USA). Protoxylem position and meristem size were estimated exploiting the Leica Application Suite Advanced Fluorescence software, and then used for statistical analysis.

### 4.3. Hydrogen Peroxide In Situ Detection

To reveal the in situ extracellular H_2_O_2_ accumulation, the fluorogenic peroxidase substrate AUR (Molecular Probes, Invitrogen, Carlsbad, CA, USA) was exploited [38], and the fluorescence of the peroxidase reaction product was detected under LSCM in root apices from 7-day-old unwounded control and leaf-wounded seedlings 6 h after injury, as hereafter described. Root apices were stained by incubation in 100 µM AUR for 5/10 min and then observed under LSCM using a 543 nm helium-neon laser with a 550–700 nm band-pass filter. For the measurement of the AUR fluorescence intensity in roots of unwounded control and leaf-wounded plants, five rectangles of approximate 65 µm^2^ for each analysed root were drawn over the protoxylem maturation zone and the sum of the pixels corresponding to the fluorescence present in each rectangle was measured exploiting the quantitative analysis of the LAS-AF software used to acquire the confocal images.

### 4.4. Statistics

The analyses under LSCM of protoxylem position, meristem size and H_2_O_2_ accumulation after PI and AUR staining, as well as root growth analysis, were performed on five independent experiments on a minimum of ten plants per treatment, yielding reproducible results. Images from single representative experiments are shown. Statistical tests of protoxylem position, meristem size and root growth were performed using GraphPad Prism (GraphPad Software, San Diego, CA, USA) with one-way ANOVA. The statistical significance of differences was evaluated by *p* levels as follows: ns, not significant; *, *p* ≤ 0.05; **, *p* ≤ 0.01; ***, *p* ≤ 0.001; and ****, *p* ≤ 0.0001. The average values of fluorescence intensity for unwounded control and leaf-wounded plants were obtained by analysing five roots for treatment, and five rectangles of approximately 65 µm^2^ for each analysed root.

## Figures and Tables

**Table 1:** Analysis of differences in root growth and meristem size in leaf-wounded seedlings grown in medium with or without the H_2_O_2_-scavenger DMTU. The effect of leaf wounding on root growth was evaluated as the difference between the length measured at the onset of the wounding and that measured after 3 days. The length of the meristematic zone was determined by measuring the distance between the quiescent centre and the first elongating cell in the cortex cell file. These experiments were repeated at least five times with ten seedlings analysed each time (mean values ± SD; *n* = 50). The statistical significance levels between unwounded control and wounded plants were evaluated by *p* levels as follows: ns, not significant.

	Root Growth (cm)		Meristem Size (μm)	
	Unwounded Control	Leaf-Wounded Plant	Unwounded Control	Leaf-Wounded Plant
**−DMTU**	2.55 ± 0.20	2.39 ± 0.15 ns	374.0 ± 28.4	372.3 ± 34.6 ns
**+DMTU**	2.54 ± 0.25	2.39 ± 0.24 ns	373.3 ± 15.9	370.1 ± 17.3 ns
